# Overcrowding in St. Pancras

**Published:** 1899-06-10

**Authors:** 


### The Hospital

A Journal of

The Medical Sciences and Hospital Administration.

Vol. XXVI.—No. 663.] Edited by Sir Henry Burdett, K.C.B., and Solomon C. Smith, M.D., M.R.C.P. [June 10, 1899.

### Overcrowding in St. Pancras.

The difficulty which has arisen in St. Pancras between the Vestry and the County Council is of widespread interest, for difficulties of just the same nature might at any moment arise in almost any of the central parishes in London. It seems that Dr. Hamer, assistant medical officer of health to the London County Council, had stated in his reports that the staff of inspectors employed by the Vestry of St. Pancras was insufficient for the proper registration and inspection of the houses let in lodgings in that parish, and had recommended that seven additional inspectors should be appointed for that and general sanitary purposes. The committee appointed to consider this report by no means agree with its conclusions, and they say that they are not prepared to recommend the Vestry to enforce the by-laws as to overcrowding strictly until such time as the London County Council shall undertake the re-housing of the people thereby disturbed. The great question underlying the whole trouble is whether the law as to overcrowding is to be enforced. Now, in discussing a subject of this importance it is necessary to take broad views, and not to limit oneself to the outlook of the vestryman dealing with the interests of his own parishioners. If one takes the merely local view, whether the vestryman looks to the improvement of the value of the property in his parish, or regards the wellbeing of its individual inhabitants, he is but doing his best for his constituents when he asks the County Council to spend money in the provision of working-men's dwellings in the parish. Again, taking it as an elementary fact, and as the basis of all argument, that a given number of people exist in the parish and must somehow be provided with house room, he is no doubt justified, from his point of view, in considering that he occupies an unassailable position when he answers all demands for the enforcement of the provisions of the Act against overcrowding by a simple *non possumus*. If, however, we put on one side petty parochial ideas, and consider the good of London as a whole—the view which of necessity the County Council has to take—we see that overcrowding is at the bottom of almost every sanitary difficulty which arises, and that unless overcrowding be dealt with in a firm and impartial manner, every sanitary improvement is apt to be nullified by its overmastering influence for evil.

Looking at the problem from the health point of view, it is not a question, as so many people seem to think it is, of how to provide for the swarming multitudes who crowd into certain areas, say of St. Pancras, but rather of how to prevent their swarming in such crowds at all. For this the building of great blocks of workmen's dwellings is no remedy. Not only is it an evil in itself, but it aggravates the very evil it is meant to relieve, in that it tends still further to make the central portions of the town the one great emporium of the labour market. What we have to remember in considering this great health problem of overcrowding is that it is not exclusively, nor even chiefly, the result of clearances for public works and sanitary improvements, but arises from the diminution of available space, owing to the steadily increasing utilisation of the central parts of London for industrial purposes. Thus at the very time when the accommodation at the disposal of the working classes in the central parts is tending to decrease, those very parts are becoming more and more the one market where alone the workman can satisfactorily dispose of his labour. Rise of rent is the result, and this, which is due to increased demand for house room as well as to diminished supply, may be taken as expressing fairly accurately the temptation to tenants to overcrowd and to landlords to encourage overcrowding.

It is needless for us in these columns to say anything about the evils of overcrowding. So great are they, and so constantly do they intrude into every sanitary question, that we have but little hesitation in asserting that but for smoke and overcrowding life in towns might be made quite as healthy as, perhaps even more so than life in the country. Whatever may be done then in the way of providing more dwellings for the working classes, all will be thrown away unless the law against overcrowding is rigidly enforced. Nor must we forget the social side of the question, or ignore the fact that such a rigid enforcement of the regulations against overcrowding is the great, and, indeed, almost the only protection which the law gives to the respectable working man against the unscrupulous competition of the sweater. We hear much nowadays about the high rents which workmen living in the central parts of London have to pay for their dwellings, and there can be no doubt of the hardship which many of them have to suffer in this regard. But rent is a relative term, and high rent would be nothing if wages rose in proportion; but they do not, and there can be no doubt that this is owing largely to the remissness of the local sanitary authorities in permitting unfair competition by those who defy the sanitary law. People may kick as they like against economic laws, but the wages in every district must in the end be fixed in accordance with the relations which exist between the supply of work, the supply of labour, and the cost of living; but the cost of living depends upon the standard of living which is permitted, for in the end a living wage has always to be paid, and, unless the State steps in, this which is the one single key to the position tends to be lowered indefinitely by unlimited competition. So strong is competition, so free is it, and so open is London as an outlet for the energies of all the world, that nothing stands between English workmen and the piggish life of the lowest of competing races but the constant and unflinching enforcement of the sanitary law, and especially the law against overcrowding. There are certain things that the State should not meddle with; there are others with which it is its duty to interfere. All experience has shown that for the State to interfere with prices, whether of food or of labour or of lodging, leads only to disaster. But it is as much the duty of the State to see that all lodgings which are let are habitable and of full capacity, as it is to see that all bread which is sold is pure and of full weight.

